# The cylindromatosis gene product, CYLD, interacts with MIB2 to regulate Notch signalling

**DOI:** 10.18632/oncotarget.2573

**Published:** 2014-11-03

**Authors:** Neil Rajan, Richard J.R. Elliott, Alice Smith, Naomi Sinclair, Sally Swift, Christopher J. Lord, Alan Ashworth

**Affiliations:** ^1^ The CRUK Gene Function Laboratory and Breakthrough Breast Cancer Research Centre, The Institute of Cancer Research, London, SW3 6JB, UK; ^2^ Institute of Genetic Medicine, Newcastle University, Newcastle upon Tyne, NE1 3BZ, UK

**Keywords:** *CYLD*, Notch, *JAG2*, *MIB2*, *RUNX1*, cylindroma, spiradenoma

## Abstract

CYLD, an ubiquitin hydrolase, has an expanding repertoire of regulatory roles in cell signalling and is dysregulated in a number of cancers. To dissect CYLD function we used a proteomics approach to identify CYLD interacting proteins and identified MIB2, an ubiquitin ligase enzyme involved in Notch signalling, as a protein which interacts with CYLD. Coexpression of CYLD and MIB2 resulted in stabilisation of MIB2 protein levels and was associated with reduced levels of JAG2, a ligand implicated in Notch signalling. Conversely, gene silencing of CYLD using siRNA, resulted in increased JAG2 expression and upregulation of Notch signalling. We investigated Notch pathway activity in skin tumours from patients with germline mutations in *CYLD* and found that JAG2 protein levels and Notch target genes were upregulated. In particular, RUNX1 was overexpressed in CYLD defective tumour cells. Finally, primary cell cultures of CYLD defective tumours demonstrated reduced viability when exposed to γ-secretase inhibitors that pharmacologically target Notch signalling. Taken together these data indicate an oncogenic dependency on Notch signalling and suggest potential novel therapeutic approaches for patients with CYLD defective tumours.

## INTRODUCTION

Ubiquitylation of proteins is a key post-translational modification and plays a role in targeting proteins for degradation *via* the proteasome as well as having roles in membrane trafficking, DNA repair, protein kinase activation and chromatin modification [1–3]. The covalent attachment of the 76 amino acid ubiquitin molecule to a protein is achieved via a cascade mediated by three enzyme classes: E1 (ubiquitin activating enzymes), E2 (ubiquitin conjugating enzymes) and E3 (ubiquitin ligases). Ubiquitylation often takes the form of polymeric ubiquitination, forming ubiquitin chains that are progressively linked through lysine-48 or lysine-63 residues. There are also five other lysine residues present in ubiquitin (K6, K11, K27, K29, K33) that are also believed to take part in atypical chain elongation [4]. Furthermore, the type of ubiquitin linkage formed is thought to determine the subsequent signal (e.g. degradation, DNA repair, receptor endocytosis). Finally, deubiquitylation of proteins performed by a set of deubiquitylating enzymes (DUBs) is considered as a crucial mechanism for cell regulation [5, 6]. DUBs and ubiquitin ligase enzymes can act as molecular partners, allowing for tight regulation of a variety of cellular process dependent on ubiquitylation [5].

The tumour repressor protein CYLD, a DUB, has previously been shown to suppress potentially oncogenic NF-κB signalling [7–9]. Loss of functional CYLD causes a disease known as Brooke-Spiegler syndrome (BSS). BSS is characterised by skin tumours that originate from hair follicles and predominantly affects the scalp and face. Specifically, BSS is caused by heritable mutations in *CYLD* that cause protein truncation and disruption of its ubiquitin-specific protease (USP) domain [10, 11]. CYLD negatively regulates NF-κB activation by removing lysine-63 linked ubiquitin chains from TRAF2/6, NEMO and BCL3, and loss of CYLD leads to hyper-activation of NF-κB signalling and an increased susceptibility to cutaneous tumour formation in mouse knockout models [7, 9, 12]. RNA interference based silencing (RNAi) of CYLD also results in activation of JNK signalling and *Cyld* deficient mice develop severe colonic inflammation, colitis-associated tumours, and hepatocellular carcinoma [13–15]. CYLD has also been shown to play a role in regulating other oncogenic signalling pathways that are relevant in skin tumours, including Wnt [16] and TGF-β signalling [17].

In haematological malignancies such as T-cell acute lymphoblastic leukaemia (T-ALL), Notch signalling regulates CYLD expression. Aberrant Notch signalling has been shown to sustain NF-κB signalling via Hes1 mediated repression of CYLD [18]. Synergistic cross-talk between Notch and NF-κB signalling has also been demonstrated in murine models of pancreatic cancer, where TNF-alpha has been shown to induce Notch target gene expression [19]. Notch signalling in development, dissected in *Drosophila* studies, is dependent on direct cellular interactions with neighbouring cells [20]. This interaction, central to processes in development such as restriction of organ size, is thought to be altered in a range of cancers, where it facilitates processes such as aberrant angiogenesis and stromal oncogenic dependency. Notch signalling has been shown to play a role (reviewed by Ranganathan *et al*. [21]) in an increasing variety of cancer types. A number of therapeutic approaches to blocking Notch signalling are being developed, including γ-secretase inhibitors.

In the minimal model of two cells communicating *via* Notch signalling, the “message-sending” cell relies upon ubiquitylation of the intracellular domain of the Delta/Serrate/LAG-2 (DSL) family to facilitate the formation of a signalling focus (microdomain) that allows interaction with the “message-receiving” cell. E3 ligases and ubiquitin hydrolases can also modulate Notch signalling by regulating levels of expression of DSL ligands by influencing their degradation [22].

Mind Bomb homologue 2 (MIB2)/skeletrophin is an E3 ligase that targets the intracellular region of the Notch family ligand, Jagged-2 (JAG2) [23]. Together with Neur and MIB, MIB2 shares a structural E3 “really interesting new gene” (RING) ligase domain that catalyses the addition of single or short chains of ubiquitin to the lysine residues that lie in the intracellular domains of DSL ligands such as JAG2. Aberrant JAG2 activity has been shown to be pathogenic in cancers such as myeloma [23], and hence dissection of its regulation might be of therapeutic importance.

Here, we report a novel interaction between CYLD and MIB2 and explore the effect of these proteins on Notch signalling. The presence of aberrant Notch signalling in skin tumours from patients with BSS further supports the hypothesis that the CYLD/MIB2 interaction might play a pathogenic role in human cancer.

## RESULTS

### CYLD interacts with MIB2

To identify novel functions of CYLD, we performed a proteomic analysis of CYLD interacting proteins [24, 25]. Briefly, this involved the co-purification of an exogenously expressed CYLD ‘bait’ protein in complex with any interacting ‘target’ protein(s), followed by SDS-PAGE based protein separation, protein extraction and subsequent identification of potential interacting proteins using mass spectrometry (see Materials and Methods).

**Figure 1:**
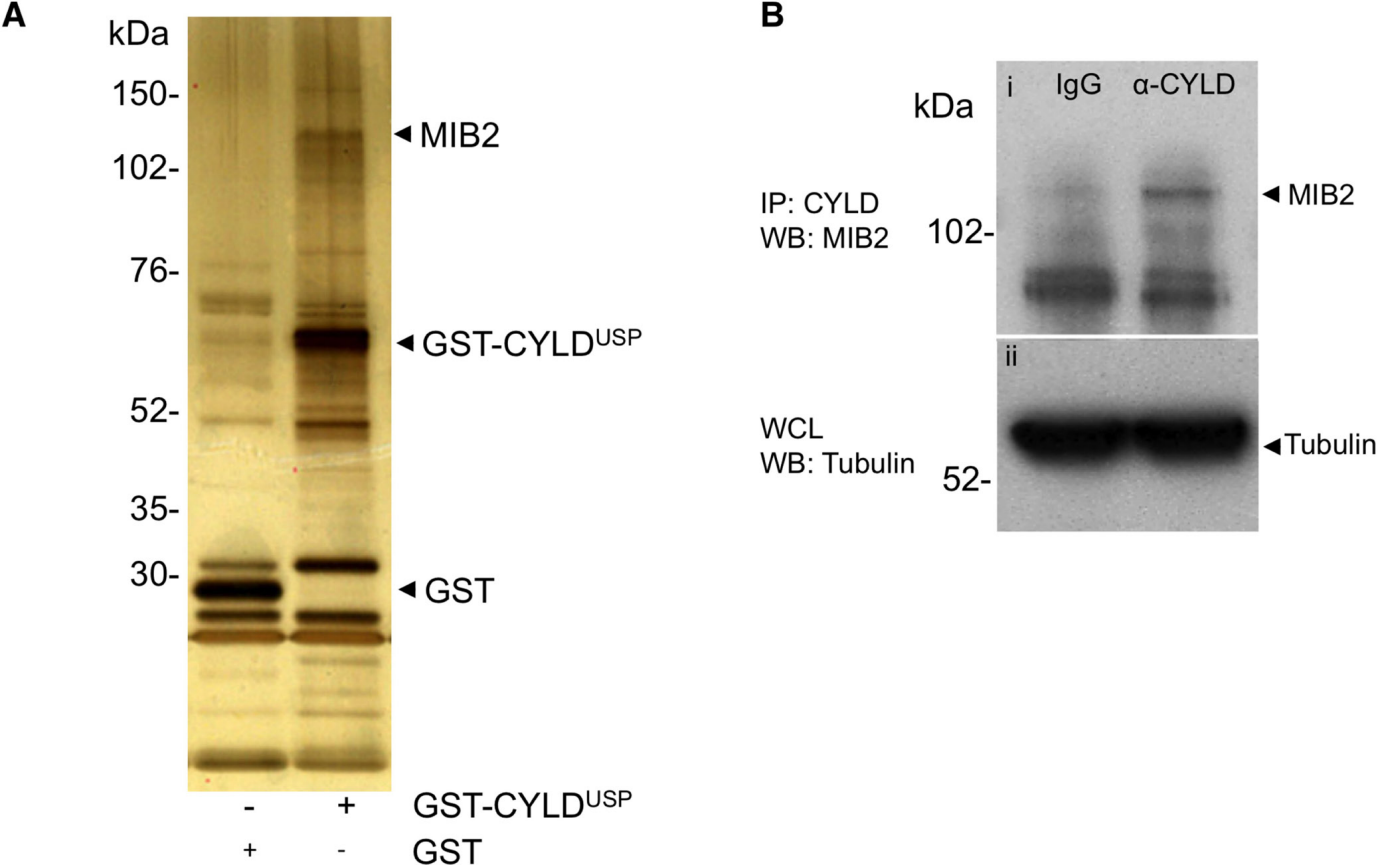
MIB2 interacts with CYLD **(A)** MIB2 interacts with CYLD in GST pull down assays. The ubiquitin hydrolase catalytic domain of CYLD (CYLD^USP^) was expressed as a fusion protein with glutathione-*S*-transferase (GST) in HEK-293T cells and CYLD-associated proteins were isolated from whole cell lysates using a GST pull down assay. MIB2 co-purified with recombinant GST-CYLD ubiquitin specific protease domain (GST-CYLD^USP^) and was identified by subjecting excised bands to tryptic digest followed by matrix assisted laser desorption and tandem mass spectrophotometry (also see Supplementary Table 1). **(B)** Endogenous immunoprecipitation of MIB2 by CYLD. Control rat IgG or rat anti-CYLD monoclonal antibody (2 μg) were incubated with HEK-293T lysates. After immunoprecipitation, the eluted proteins were analysed by western blot using a rabbit anti-MIB2 polyclonal antibody. MIB2 is immunoprecipitated by CYLD and not IgG control (panel i). Tubulin expression was used as a loading control (panel ii).

The ubiquitin hydrolase domain of CYLD (CYLD^USP^- amino acid residues 583-956), which was used as a ‘bait’ protein (To detect interacting proteins with the catalytic portion of CYLD), was expressed as a fusion protein with glutathione-*S*-transferase (GST) following transfection into HEK-293T cells. TNF-alpha was added to the media to stimulate NF-κB signalling. CYLD-associated proteins from cells expressing CYLD^USP^ were isolated using a GST-based precipitation assay. Whole cell lysates generated from transfected cells were incubated with glutathione sepharose and the resin bound complex(es) copiously washed with buffer to remove non-specifically binding contaminants. To control for non-specific binding effects mediated by the GST tag, this analysis was also carried out in cells expressing the GST tag alone. GST-CYLD^USP^ and possible binding partners were then electrophoresed on PAGE gels and visualised by silver staining (Figure 1A). Prominent bands associated with the expression of GST-CYLD^USP^ but not GST alone were then excised and subject to tryptic digestion. Matrix Assisted Laser Desorption-TOF-TOF mass spectrometry was then used to identify these digested products and bioinformatic identification using fragment ion search was carried out using the ProFound search engine (Rockefeller University) [26].

Amongst the bands analysed by mass spectrometry, MIB2 was identified with reasonable probability as a CYLD interacting protein (*P* = 81%) and 12 different MIB2 peptides, representing 13% of the total MIB2 amino acid sequence, were identified (Supplementary Table 1). MIB2 is an E3 ubiquitin ligase associated with the Notch pathway and its interaction with an ubiquitin hydrolase, CYLD, raised the possibility that this interaction may highlight a novel mechanism of regulating Notch signalling. Therefore, the CYLD/MIB2 interaction was investigated in more detail. The interaction of CYLD-MIB2 was seen both in unstimulated and TNF-alpha stimulated cells, and subsequent experiments were performed without TNF-alpha stimulation (Supplementary Figure 1). The interaction between endogenous CYLD and MIB2 was validated by co-immunoprecipitation of CYLD with MIB2 (Figure 1B). This experiment was performed in the presence of the proteasome inhibitor MG-132, which reduced the rate of degradation of MIB2-Ubiqitin species, allowing this interaction to be more easily detected. The potential for a functional interaction between CYLD and MIB2 was investigated further.

### Characterisation of the CYLD/MIB2 interaction

**Figure 2:**
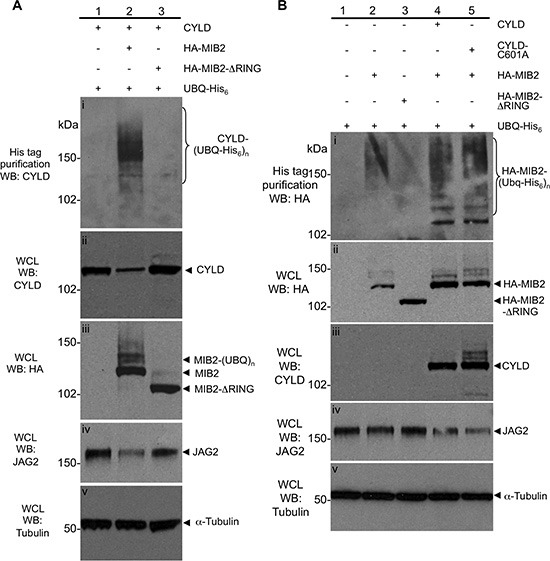
Characterisation of the ubiquitylation status and protein levels of CYLD and MIB2 **(A)** MIB2 ubiquitylates CYLD, resulting in its degradation and a reduction in levels of Notch ligand JAG2. HA-MIB2, CYLD (untagged) and His_6_-ubiquitin constructs were co transfected in HEK-293T cells and drove the ubiquitylation of CYLD compared to cells transfected with CYLD/His_6_-ubiquitin alone (panel (i), compare lanes 1 and 2). Co-transfection of CYLD/HA-MIB2-ΔRING/His_6_-ubiquitin prevented auto-ubiquitylation of MIB2 (panel (iii), compare lanes 2 and 3) and did not result in ubiquitylation of CYLD (panel (i), compare lanes 2 and 3). Overexpression of MIB2 but not MIB2-ΔRING appeared to decrease endogenous levels of its downstream target, JAG2 (panel (iv)). Tubulin was used as a loading control (panel (v)). **(B)** CYLD expression is associated with increased ubiquitylated MIB2 protein and reduced JAG2 protein, independent of its ubiquitin hydrolase activity. Lysates from transfected cells prepared in denaturing buffer were analysed by western blot and purified by nickel chromatography and showed that in the absence of exogenous CYLD expression, overexpression of HA-MIB2/His_6_-ubiquitin predominantly resulted in high order molecular weight (MW) products at around 150 kDa (panel (i), lane 2) and decreased MIB2 in the whole cell lysate (panel (ii), lane 2 compared to lane 4 and 5). In the presence of exogenous CYLD expression, a larger proportion of lower MW ubiquitylated MIB2 products were observed (panel (i) compare lane 2 *vs*. lane 4). In order to determine whether this was dependent on CYLD deubiquitylase activity, HA-MIB2/His_6_-ubiquitin was co-expressed with a catalytically inactive CYLD (CYLD-C601A, with no apparent difference in poly-ubiquitylated MIB2 levels versus co-expression with wild type CYLD seen (panel (i), compare lane 4, 5 and MIB2 levels in whole cell lysate, panel (ii), lanes 2, 4 and 5). Tubulin was used as a loading control (panel (v)).

MIB2 is an E3 ligase, known to ubiquitylate itself and the intracellular region of JAG2, which is a single pass transmembrane protein [23]. To further dissect the nature of the CYLD/MIB2 interaction, we performed ubiquitylation assays, which allowed us to quantify the expression of both proteins and respective ubiquitylated species when catalytically active and inactive CYLD and MIB2 were expressed. These assays were performed by transiently co-transfecting HEK-293T cells with cDNA constructs containing the open reading frame of either CYLD, MIB2 or both in combination, together with a construct expressing polyhistidine tagged ubiquitin (His_6_-ubiquitin). The expression of His_6_-ubiquitin allowed us to analyse ubiquitin conjugated proteins following capture by nickel affinity chromatography under denaturing conditions. First, co-transfection of haemagglutinin epitope tagged-MIB2 (HA-MIB2) [27], CYLD (untagged) and His_6_-ubiquitin expression constructs clearly drove the ubiquitylation of CYLD compared to cells transfected with CYLD and His_6_-ubiquitin (Figure 2A panel (i), compare lanes 1 and 2), suggesting that MIB2 may ubiquitylate CYLD. To test whether loss of MIB2 E3 ligase activity mediated these effects, the C-terminus of the HA-MIB2 construct was mutated so that it expressed MIB2 without the catalytic RING domains (HA-MIB2-ΔRING). Co-transfection of CYLD/HA-MIB2-ΔRING/His_6_-ubiquitin prevented auto-ubiquitylation of MIB2 (Figure 2A, panel (iii), compare lanes 2 and 3) and did not result in ubiquitylation of CYLD (Figure 2A panel (i), compare lanes 2 and 3). The latter observation suggested that MIB2 catalytic activity could be responsible for the ubiquitylation of CYLD. Furthermore, overexpression of MIB2 but not MIB2-ΔRING appeared to decrease endogenous levels of its downstream target, JAG2 (Figure 2A, panel (iv)), presumably by promoting its endocytosis and degradation, which is mechanistically consistent with previous observations [23]. Taken together, these observations suggested that MIB2 ubiquitylates CYLD leading to CYLD destabilisation and degradation, and that CYLD might be involved in regulation of JAG2, and ultimately Notch signalling, via its interaction with MIB2.

As we demonstrated that MIB2 can ubiquitylate itself as well as CYLD, we postulated that CYLD, as an ubiquitin hydrolase, might deubiquitylate MIB2, hence providing a potential mechanism of mutual regulation. To test this hypothesis we assessed the effect of CYLD catalytic activity upon MIB2 ubiquitylation levels. Cell lysates prepared in denaturing buffer were purified by nickel chromatography and analysed by western blotting. This demonstrated that in the absence of exogenous CYLD expression, overexpression of HA-MIB2/His_6_-ubiquitin predominantly resulted in high order molecular weight (MW) MIB2 ubiquitylated products at around 150 kDa (Figure 2B panel (i), lane 2) and decreased MIB2 expression in the whole cell lysate (Figure 2B, panel (ii), lane 2 compared to lane 4 and 5). However, in the presence of exogenous CYLD expression, a larger proportion of lower MW ubiquitylated MIB2 products were observed (Figure 2B panel (i) compare lane 2 *vs*. lane 4). This suggests that CYLD may play a role in MIB2 regulation and subsequent Notch signalling, as in this case we appear to observe that overexpression of CYLD causes a stoichiometric imbalance of high- and low-molecular weight MIB2-(Ubiquitin)_n_ species. Inefficient MIB2 processing, which is normally carried out *via* proteasomal degradation of higher molecular weight MIB2-(Ubiquitin)_n_ species [28], could then lead to dysregulation of JAG2 levels and, consequently, the downstream Notch signal.

In order to determine whether this was dependent on CYLD deubiquitylase activity, HA-MIB2/His_6_-ubiquitin was co-expressed with a catalytically inactive CYLD mutant cDNA (CYLD-C601A, [9]). However we observed no apparent difference in poly-ubiquitylated MIB2 levels in CYLD-C601A expressing cells compared to wild-type CYLD expressing cells (Figure 2B, panel (i), compare lane 4, 5 and MIB2 levels in whole cell lysate, panel (ii), lanes 2, 4 and 5).

Taken together these results suggested that CYLD interacts with and is ubiquitylated by MIB2. However likely MIB2 is not a deubiquitylation target of CYLD, but rather is stabilised by CYLD in a non-catalytic dependent manner. The MIB2 target, JAG2, also did not appear to be a deubiquitylation target of CYLD (data not shown). The observed stabilisation of MIB2 by CYLD appeared to effect downstream degradation of endogenous JAG2. (Figure 2B, panel (iv), lanes 4 and 5).

### CYLD depletion activates Notch signalling

**Figure 3:**
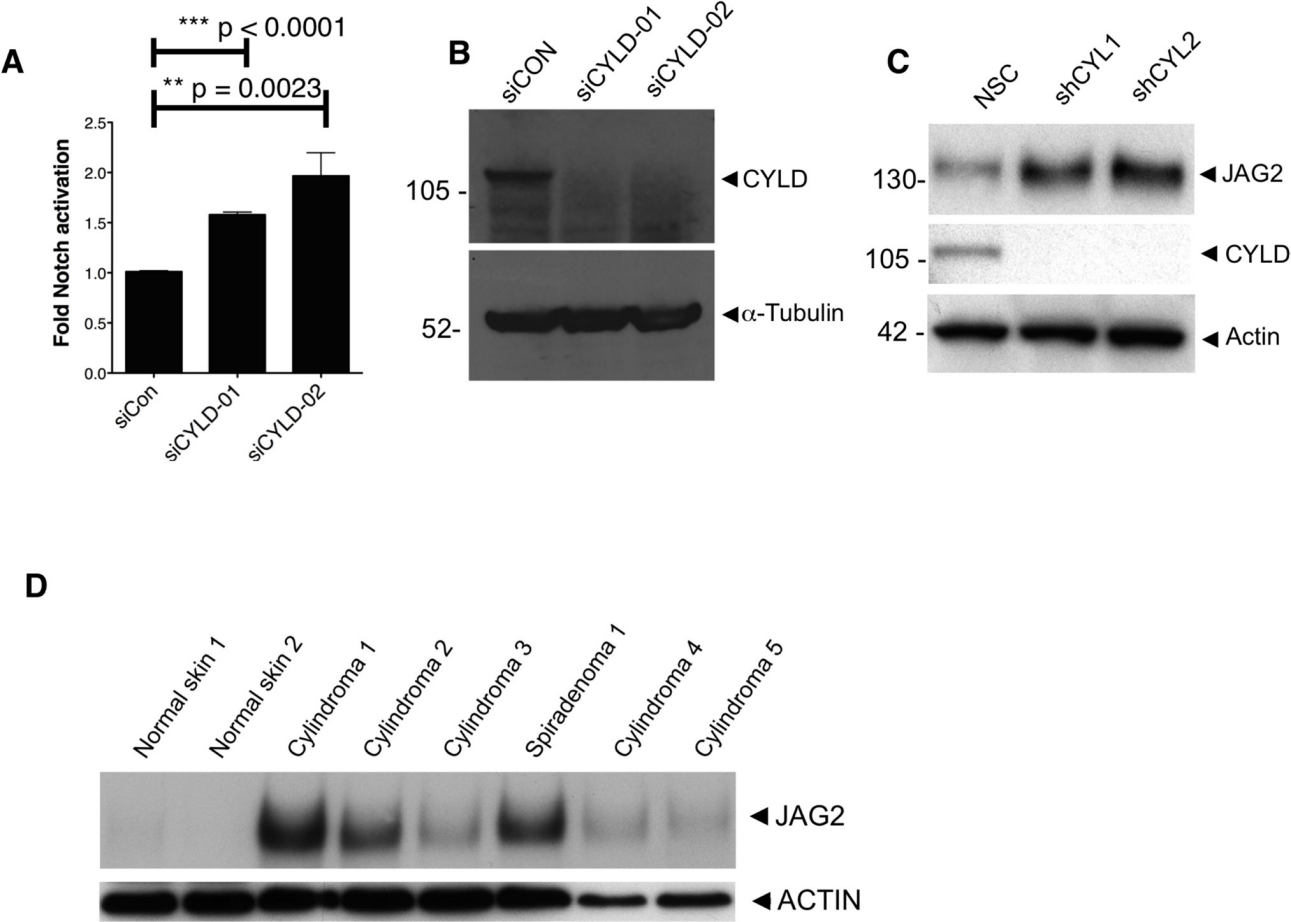
CYLD ablation results in increased Notch signalling **(A)** HEK-293T cells stably expressing a RBP-JK response element coupled to a firefly luciferase were used as a readout for active Notch signalling. Notch signalling was significantly increased following CYLD knockdown with siRNA targeting CYLD (unpaired t-test). **(B)** CYLD protein expression reduction following siRNA targeting was demonstrated by probing lysates from transfected cells with anti-CYLD antibody **(C)** CYLD protein expression reduction is associated with increased JAG2 expression, and **(D)** increased JAG2 expression is seen in the majority of CYLD defective tumour lysates obtained from patients with germline mutations in *CYLD.*

We assessed the potential role of the CYLD/MIB2 interaction with respect to Notch signalling. CYLD depletion using siRNA is known to activate NF-κB signalling [9], and we therefore investigated the effect this had on the Notch pathway. CYLD siRNAs were transfected into cells that had been stably transduced with a lentiviral luciferase reporter under the control of the RBP-JK transcriptional response element, which served as a readout of Notch signalling. Seventy-two hours post-transfection, the cell lysates were assayed for RBP-JK related luciferase activity (Figure 3A). Notch activation in CYLD siRNA transfected cells was normalised to cells transfected with non-silencing siRNA. This analysis showed that Notch activity significantly increased ( p < 0.01 - unpaired t-test) in response to gene silencing of CYLD; efficient CYLD silencing by the siRNA was confirmed by western blot analysis (Figure 3B). Increased JAG2 expression was also demonstrated in 293 cells with CYLD silencing (Figure 3C). To determine if this finding was relevant in a human model, we assessed JAG2 expression in CYLD defective tumour lysates, obtained from patients with germline *CYLD* mutations. These consisted of 5 cylindromas and 1 spiradenoma, obtained from 4 patients. Normal skin exhibited undetectable levels of JAG2 compared to 5 cylindromas and one spiradenoma, even when enhanced amounts of normal skin protein were assessed in western blots (Figure 3D).

### Notch target genes are dysregulated in CYLD defective tumours

**Figure 4:**
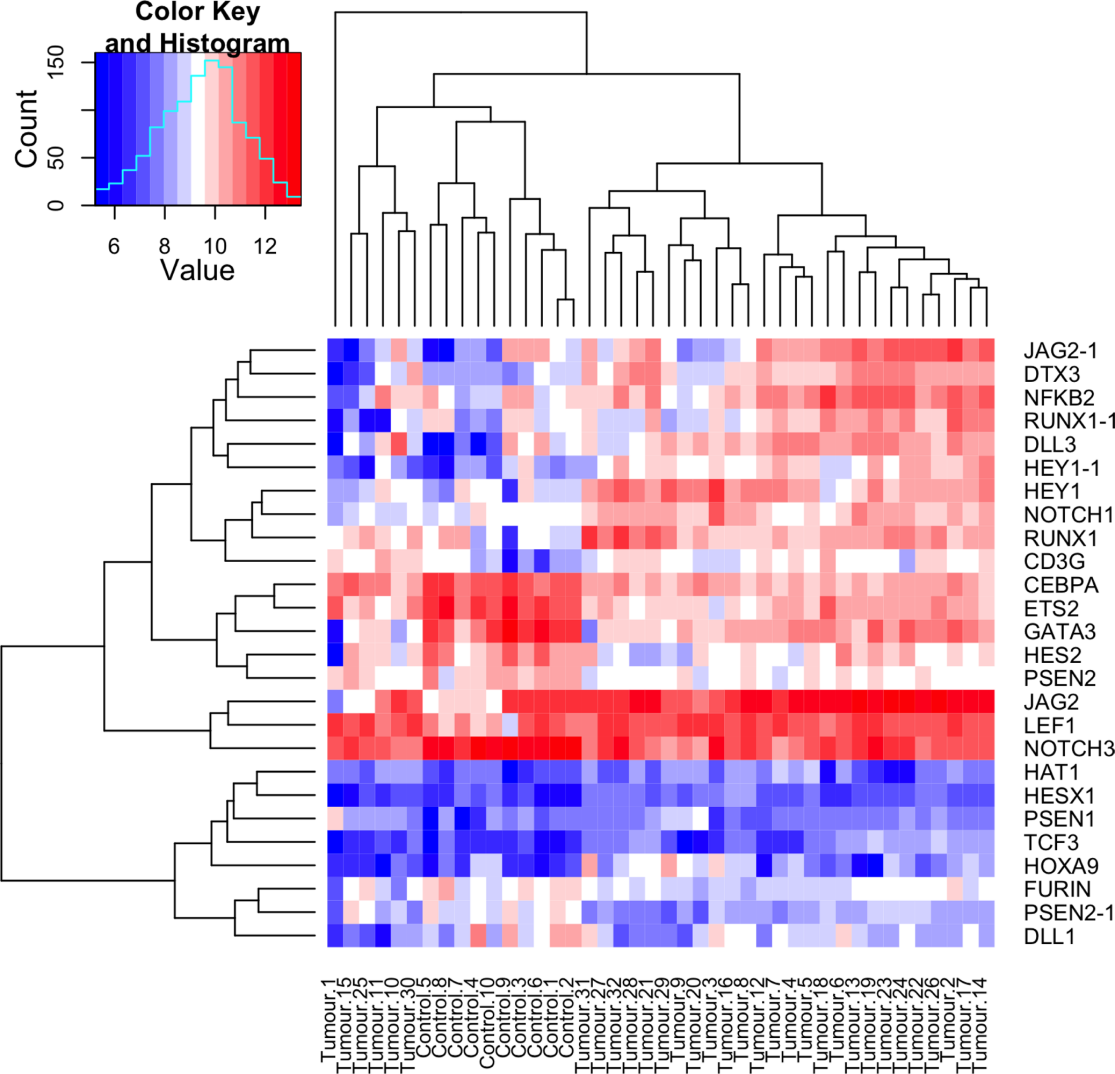
Notch target genes and members of the Notch signalling pathway are overexpressed in CYLD defective tumours A heatmap plot illustrating the expression levels of Notch signalling pathway and target genes in 32 CYLD defective tumours and 10 controls. Notch target genes and signalling pathway members that were differentially expressed with a p value of <0.05 across an average of 32 tumours compared to an average of 10 controls (tumours and controls are indicated at the bottom of the heatmap) were included, and transcript expression levels at a single sample level are illustrated in the heatmap. Overexpressed transcripts are shown in red and transcripts that are underexpressed are shown in blue, with gene names indicated on the right hand side of the figure. Where data from multiple transcripts for a single gene were available, these are indicated with a numerical suffix (n = 4). Clustering by similarity is shown as determined by Euclidean distance.

Given the increase in luciferase activity indicative of Notch activity in cells transfected with CYLD siRNA, we explored the consequences of *CYLD* inactivation in skin tumours from patients with germline mutations in *CYLD*. This model was used to determine if features associated with increased Notch signalling such as the expression of Notch target genes were demonstrated in a human CYLD defective model, as suggested by the luciferase assay. We assessed the expression of 98 Notch related transcripts, including Notch target genes and Notch signalling pathway components [29], in a dataset of gene expression profiles of CYLD defective tumours [30]. Genes that were differentially expressed in the pooled signature of 32 CYLD defective tumours compared to 10 controls with a threshold for statistical significance of p < 0.05 after correction for multiple hypothesis testing were included (n = 26). The fold changes of these genes are detailed in Table 1. To visualise the expression of each of these genes at an individual sample level, normalised signal values were plotted on a heatmap following logarithmic transformation (Figure 4). Clustering of this data by Euclidean distance clearly differentiated the tumours from the control tissue. Six tumours (Tumour numbers: 1, 10, 11, 15, 25, 30), of which four were trichoepitheliomas in the study set clustered distinctly from the majority of cylindroma and spiradenomas (n = 28). A number of Notch target genes were consistently overexpressed in CYLD defective tumours, including *LEF1, HEY1 and NOTCH1*. The increased expression of *JAG2* in CYLD defective tumours in particular supported our *in vitro* findings that CYLD-MIB2 regulation of Notch signalling influences expression levels of the ligand JAG2.

**Table 1:** Notch target genes are upregulated in CYLD defective tumours Gene expression data from 32 cylindroma tumours and 10 control perilesional skin samples were pooled into tumour and control groups. Differentially expressed genes with a threshold of p < 0.05 were included and the fold changes (FC) are indicate for the respective transcripts.

Gene	FC	ILMN Probe ID	Description
DTX3	3.13	ILMN_1658677	Deltex homolog 3 (Drosophila)
HEY1	2.73	ILMN_1788203	Hairy/enhancer-of-split related with YRPW motif 1
JAG2	2.50	ILMN_2399523	Jagged 2
HEY1	2.43	ILMN_2407308	Hairy/enhancer-of-split related with YRPW motif 1
DLL3	2.40	ILMN_1736096	Delta-like 3 (Drosophila)
RUNX1	2.33	ILMN_1801504	Runt-related transcription factor 1
HOXA9	2.19	ILMN_1739582	Homeobox A9
TCF3	2.09	ILMN_1664434	Transcription factor 3 (E2A immunoglobulin enhancer binding factors E12/E47)
NFKB2	1.91	ILMN_1799062	Nuclear factor of kappa light polypeptide gene enhancer in B-cells 2 (p49/p100)
CD3G	1.88	ILMN_1717197	CD3g molecule, gamma (CD3-TCR complex)
JAG2	1.87	ILMN_1764729	Jagged 2
LEF1	1.80	ILMN_2213136	Lymphoid enhancer-binding factor 1
NOTCH1	1.67	ILMN_1729161	Notch homolog 1, translocation-associated (Drosophila)
HAT1	1.65	ILMN_1693905	Histone acetyltransferase type B catalytic subunit
PSEN1	1.56	ILMN_1809193	Presenilin 1
RUNX1	1.54	ILMN_1669335	Runt-related transcription factor 1
HESX1	1.38	ILMN_1742929	HESX homeobox 1
FURIN	0.68	ILMN_1790228	Furin (paired basic amino acid cleaving enzyme)
PSEN2	0.61	ILMN_1714417	Presenilin 2 (Alzheimer disease 4)
PSEN2	0.49	ILMN_2404512	Presenilin 2 (Alzheimer disease 4)
DLL1	0.45	ILMN_1743373	Delta-like 1 (Drosophila)
CEBPA	0.44	ILMN_1715715	CCAAT/enhancer binding protein (C/EBP), alpha
NOTCH3	0.43	ILMN_1658926	Notch homolog 3 (Drosophila)
HES2	0.43	ILMN_2094266	Hairy and enhancer of split 2 (Drosophila)
GATA3	0.34	ILMN_2406656	GATA binding protein 3
ETS2	0.32	ILMN_1720158	V-ets erythroblastosis virus E26 oncogene homolog 2 (avian)

To validate these findings, we performed quantitative PCR on RNA isolated from a subset of CYLD defective tumours used in the microarray study. Expression of *HEY1* transcript was assessed in 15 CYLD defective tumours (Supplementary Figure 2). RNA was extracted from microdissected, snap-frozen tumour tissue and subject to quantitative PCR using probes for *HEY1*. Beta-actin expression was also assessed and used to normalise expression levels. Differential expression was calculated using the 2^(−ΔΔCt)^ formula as amplification efficiencies were similar. This demonstrated upregulation of the Notch target gene *HEY1* in all specimens assayed, consistent with the findings from the microarray data.

### Immunohistochemical assessment of the expression of Notch target genes in CYLD defective tumours

**Figure 5:**
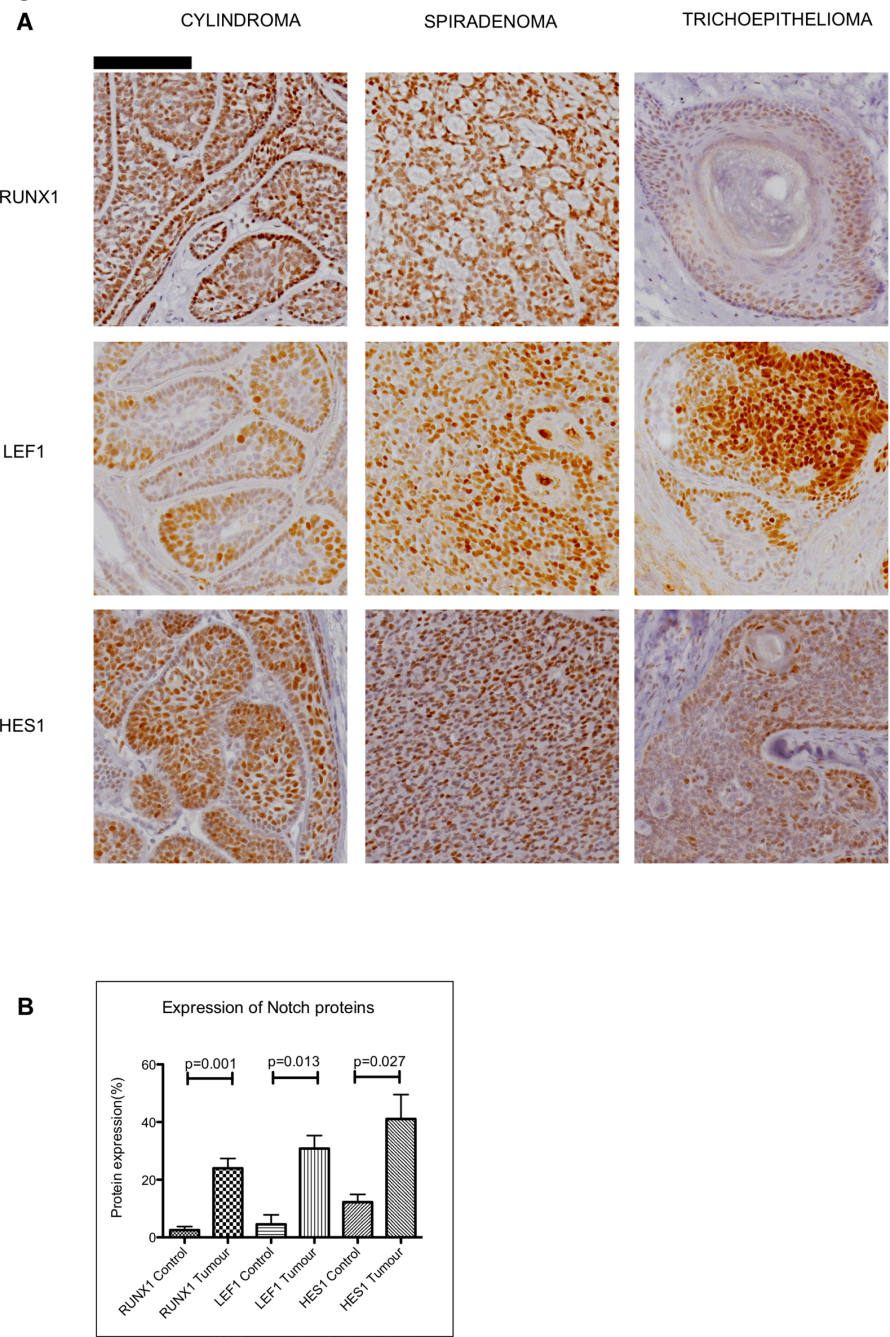
Expression of Notch target genes in CYLD defective tumours **(A)** Tissue microarrays of cylindromas and spiradenomas and perilesional control skin were probed with anti-RUNX1, anti-LEF1 and anti–HES1 antibody and protein expression was visualized with a horseradish peroxidase/3,3′-Diaminobenzidine (DAB) system, with haematoxylin used as a nuclear counterstain. Nuclear staining of LEF1, RUNX1 and HES1 was seen in CYLD defective tumours. Scale bars indicate 100μm. **(B)** Notch target proteins are increased in CYLD defective tumours when compared to perilesional control skin. Photomicrographs of tumour sections and control tissue were subject to quantitation of DAB staining as a readout of protein expression. This demonstrated an increase in expression of LEF1 ( *p* = 0.001), RUNX1 ( *p* = 0.013) and HES1 ( *p* = 0.027) in CYLD defective tumours when compared to control tissue. All p-values indicated were derived using a t-test. Error bars indicate standard error of the mean

To determine whether the observations described above seen in mRNA were reflected in changes in protein expression, we assessed protein expression of selected upregulated Notch target genes in CYLD defective tumours, and adjacent normal epidermis was used as a control. Strong nuclear staining of protein from both Notch target genes *LEF1* and *RUNX1* were observed in cylindromas, spiradenomas and trichoepitheliomas (Figure 5a). Assessment of protein expression by measurement of immunostaining intensity using Image J software showed this effect to be statistically significant when compared to adjacent normal epidermis (Figure 5b - LEF 1 p = 0.013; RUNX1 p = 0.001). We also examined expression of *HES1*, a canonical Notch target gene not highlighted by the microarray data. Nuclear HES1 was seen to be increased in CYLD defective tumours, but the difference from control epidermis was smaller than for LEF1 and RUNX1 (p = 0.027). Taken together, these data validate the microarray data at the protein level and highlight CYLD defective tumours as a model tumour type characterised by aberrant Notch signalling.

### Primary cultures of CYLD defective tumours are sensitive to γ-secretase inhibitors

**Figure 6:**
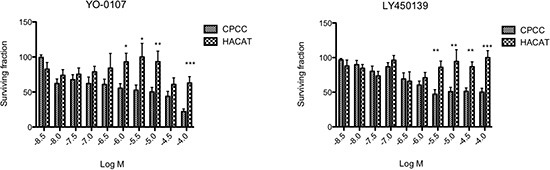
Primary cultures of CYLD defective tumours are sensitive to γ-secretase inhibitors Primary cells (CPCC) derived from fresh tumour tissue obtained following surgical procedures on *CYLD* mutation carriers or a keratinocyte cell line (HACAT) were cultured on 3D tissue culture scaffolds. After 28 days, scaffolds were grown in the presence of two different γ-secretase inhibitors (YO-0107 and LY 450139) for 12 days in triplicate and cell viability was assessed using an ATP dependent luminescent assay (Cell-Titre Glo – Promega UK). This was repeated using at least three different tumours, from different body sites in three patients. Data displayed represents the mean of 4 biological replicates of CPCC, with 3 technical replicates each. Cylindroma cells demonstrated differential sensitivity to inhibition with γ-secretase inhibitors at low micromolar concentrations of YO-0107 and LY 450139. Error bars indicate standard error of the mean. *p* < 0.05 (*), *p* < 0.01 (**) and *p* < 0.001 (***) as indicated, calculated using a t-test.

γ-Secretase inhibitors suppress Notch signalling by impairing cleavage and release of the intracellular domain of Notch protein (NICD). A reduction in free NICD results in its reduced nuclear translocation and binding to the RBP-JK complex, with a consequent reduction in transcription of Notch target genes[31]. To determine if CYLD defective tumours exhibited an oncogenic dependency on Notch signalling, primary cells derived from these tumours were cultured on 3D scaffolds as previously described [30]. Briefly, these are primary cells derived from explant culture of CYLD defective tumours removed from patients with known germline mutations in *CYLD*. These cells were obtained immediately following tumour-burden reductive surgery. Cells were grown on 3D tissue culture scaffolds and allowed to mature over 28 days, before they were cultured in varying concentrations of γ-secretase inhibitors. These CYLD defective primary cultures demonstrated sensitivity to γ-secretase inhibition at micromolar concentrations (Figure 6). A similar sensitivity was not seen in controls of a keratinocyte cell line (HaCAT) grown in identical conditions.

## DISCUSSION

Here we report a novel interaction between an ubiquitin hydrolase, CYLD, and an ubiquitin ligase, MIB2, and its consequences on Notch signalling. Ubiquitylation serves as a regulatory process in multiple cell signalling cascades, which underscores the significance of a novel ubiquitin hydrolase-ubiquitin ligase interaction. Deubiquitylases and E3 ligases frequently interact [32]. CYLD and ITCH (an ubiquitin ligase) are one such pair, interacting to regulate inflammation by modifying the ubiquitylation status of TAK1 and hence modulating NF-κB signalling [33]. The effect of such an interaction may regulate E3 ligase autoubiquitylation [34], confer specificity between the DUB and substrate [35] or allow the E3 ligase to regulate its target as well as its DUB simultaneously [36].

**Figure 7:**
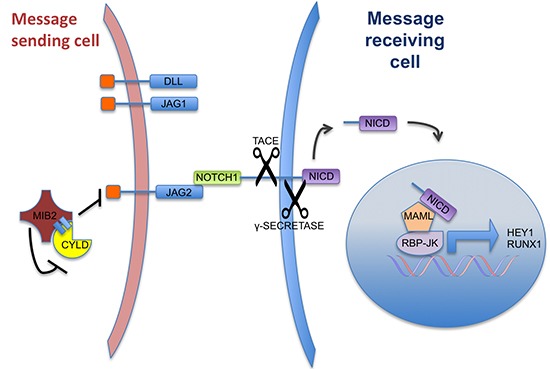
A schematic representation of Notch signalling and the suggested role of CYLD Notch signalling between 2 cells that is mediated by JAG2-NOTCH interaction. The CYLD-MIB2 complex negatively regulates JAG2 expression levels, resulting in a reduction of Notch activation. CYLD knockdown or truncation results in increased JAG2 expression Notch target gene activation (such as RUNX1) in adjacent cells.

MIB2, has been shown to modulate Notch signalling [23]. MIB2 binds to the intracellular domain of JAG2, but not other Notch ligands such as JAG1 or DLL. Ubiquitination of intracellular JAG2 is thought to increase the effectiveness of Notch signalling, and is associated with increased expression of Notch target gene HES1. Mechanistically, this is thought to be due to MIB2 mediated endocytosis of JAG2 in the message sending cell which is important for Notch ligand activation in the message receiving cell [37]. Our data suggests that the CYLD/MIB2 interaction may attenuate Notch signalling by reducing levels of JAG2 (Figure 7). In our overexpression studies, reduction of JAG2 appears to be independent of the catalytic activity of CYLD, and may be due to stabilisation of MIB2. Given the specificity of CYLD for K63-ubiquitin linked chains [38], our data would suggest that (1) auto-ubiquitylation of MIB2 is most likely a K48-ubiquitin event leading to its proteasomal degradation (in agreement with [23]); (2) the CYLD-MIB2 protein-protein interaction physically prevents efficient auto-ubiquitylation and degradation of MIB2; and (3) MIB2 regulates this process by targeting CYLD for degradation.

The CYLD defective tumours described here provide a human skin tumour model to explore the effects of truncating *CYLD* mutations on Notch signalling in a disease-relevant cellular context. These tumours have been characterised previously and frequently demonstrate loss of heterozygosity at the locus of *CYLD* [30]. We have previously shown that JAG2 is upregulated in CYLD defective tumour tissue, particularly in “Wnt active” tumours [39]. Here, we demonstrate that recognised Notch target genes are upregulated in this model, including *RUNX1, LEF1, and HES1*. Whilst some Notch genes were consistently upregulated, others varied between tumours. At the protein level, RUNX1 expression in cylindroma has recently been highlighted by Scheike *et al.* [20], and our data extends the expression of RUNX1 to include spiradenoma and trichoepithelioma, suggesting that this is a feature consistent across CYLD defective tumours. RUNX1 expression has been shown to be oncogenic in murine models [40], exerted via downstream effects on STAT3 signalling. Taken together with our data showing CYLD defective tumours are sensitive to γ-secretase inhibitors, an oncogenic dependency on Notch is likely in this model. Our data highlights a novel interacting partner of CYLD, MIB2, that influences Notch signalling and furthermore the potential therapeutic utility of γ-secretase inhibition in CYLD defective tumours.

## MATERIALS AND METHODS

### Co-purification of CYLD-protein complexes and MS identification

HEK-293T cells (5 × 10 cm dishes, seeded at 2 ×10^6^ cells) were transfected with pEBG-Empty and pEBG-CYLD^USP^ (amino acids 583-956). After 24 hours, the cells were split 1:2. After 48 hours the appropriate cells were treated with TNFα (50 ng/mL) for six hours. The cells were then chilled on ice, washed with cold PBS and lysed in cold lysis buffer for 30 minutes on ice (lysis buffer: 50 mM Tris pH 8, 150 mM NaCl, 1% Triton X-100, 10% glycerol, 1 mM EDTA, 1 mM DTT, 2 mM Benzamidine, 0.5 mM PMSF, protease inhibitor cocktail (Roche), 50 mM NaF, 1mM Na_3_VO_4_, 15 mM Na_4_P_2_O_7_). The lysates were then centrifuged and quantified (Bradford assay, Bio-Rad protein assay reagent). Glutathione sepharose (100 μL) was washed three times with lysis buffer before adding 5 mg of total lysate, with the final volume made up to 500 μL with lysis buffer. The mixtures were then incubated for 2 hours at 4^o^C on a turn disc. The resin was then collected by centrifugation, washed four times with wash buffer A (50 mM Tris pH 8, 150 mM NaCl, 0.1% Triton X-100, 10% glycerol, 1 mM EDTA, 1 mM DTT, protease inhibitor cocktail (Roche), 50 mM NaF, 1mM Na_3_VO_4_, 15 mM Na_4_P_2_O_7_) and twice with wash buffer B (50 mM Tris pH 8, 500 mM NaCl, 0.1% Triton X-100, 10% glycerol, 1 mM EDTA, 1 mM DTT). The resin was then incubated with 300 μL elution buffer (50 mM Tris pH 8, 500 mM NaCl, 20 mM reduced glutathione, 1 mM EDTA) for 20 minutes. The supernatant was then collected, 45 μL of 100% trichloroacetic acid was added and the sample mixed by vortex, followed by incubation at 4^o^C for 18 hours. The pellet was collected by centrifugation, washed with cold acetone, dried by speedi-vac and resuspended in SDS sample loading buffer. Residual TCA was neutralised by the addition of 1M Tris pH 10. The samples were then loaded onto a 4–12% NuPAGE Bis-Tris gradient gel (Invitrogen) and run at 200 V for 1 hour. Protein bands were visualised by silver stain (SilverQuest™, Invitrogen) and bands of interest excised and identified by in-gel tryptic digest, Matrix Assisted Laser Desorption-TOF-TOF (TopLab GmbH, www.toplab.de, Proteomics analyser 4700) and peptide mass fingerprint (database search: GPS-Explorer/Mascot). MIB2/skeletrophin was the top ranked ID with 13% minimum sequence coverage.

### Endogenous co-immunoprecipitation assay

293T cells (2x T175 flasks) were incubated with MG-132 (5 uM) for six hours without TNF-alpha. The cells were then washed with cold PBS and lysed in a total of 5 mL Lysis buffer (50 mM Tris pH 8, 250 mM NaCl, 0.5% NP-40, 2 mM DTT, 0.5 uM MG-132). The lysate was pre-cleared with Protein A/G sepharose (Santa Cruz) for 1 hour at 4^o^C on a turn disc, the sepharose was discarded and the lysate quantified and divided into two mixtures, A & B (8 mg total protein each). Some lysate was retained for whole cell lysate western blot control. To mixture A was added Rat anti-CYLD antibody (5 ug), to mixture B was added normal Rat IgG (5 ug) followed by incubation at 4^o^C overnight on a turn disc. Protein A/G sepharose (50 uL bed volume) was washed in lysis buffer and then added to the lysate-antibody mixtures. After incubation for 4 hours at 4^o^C on a turn disc, the resin was collected and washed copiously with lysis buffer. PAGE sample loading buffer was then added to the resin, the samples boiled and loaded onto 4–12% Bis-Tris gel alongside whole cell lysate control (100 ug). The electro-blotted membrane was then probed with anti-MIB2 antibody (1:500).

### Tumour samples

Tumour tissue was obtained from patients undergoing surgery indicated to control tumour burden or for symptomatic relief, under regional ethical committee approval (REC REF:06/1001/59).

### Plasmids, cell lines and reagents

Full length, untagged CYLD was sub-cloned into pcDNA-3.1 from FLAG-CYLD [9]. CYLD-C601A was obtained by site directed mutagenesis (Stratagene) of pcDNA-CYLD (1801-TGT>GCT) GST-CYLD^USP^ was obtained by PCR amplification of the CYLD USP domain and cloning into the pEBG vector. HA-MIB2 was a kind gift from Tamotsu Takeuchi, Kochi, Japan. HA-MIB2-ΔRING was obtained by site directed mutagenesis of HA-MIB2 via creation of a STOP codon at residue 2829 (TGC>TGA). pCW7-[Ubiquitin-His_6_] was obtained from R. Kopito, Stanford (via the ATCC/LGC Standards, MBA-85). Fugene 6 was purchased from Promega. HEK293T Notch reporter cells were obtained via use of Cignal RBP-Jk Reporter (luc) Kit: CCS-014L (SABiosciences, UK.). All cell lines were incubated at 37^o^C and 5% CO_2_ in Dulbecco's Modified Eagle Medium (GIBCO) with 10% Fetal Bovine Serum (GIBCO). All cell lines were monitored for mycoplasma infection. Anti-HA antibody (Roche), anti-JAG2 antibody (New England Biolabs), anti-MIB2 antibody (Cambridge Bioscience Ltd) were used as per the manufacturers instructions. Monoclonal Rat anti-CYLD antibody was raised against the USP domain of recombinant CYLD protein and probed at 1;500 in 5% non-fat milk. HRP-conjugated secondary antibodies (Pierce) were used at 1:5000 in 5% non-fat milk. MG-132 and recombinant TNFα was obtained from Sigma Aldrich. Duplex siRNA pools (siGenome) targeting CYLD were obtained from OpenBiosystems. Nickel Sepharose 6 was obtained from GE Healthcare.

### Ubiquitylation assays

HEK293T cells were transfected (Fugene 6) in 10cm dishes with 6ug total DNA of the indicated constructs at 1:2:3 ratio (CYLD 1μg, Ubiquitin-His_6_ 2μg, MIB2/MIB2-ΔRING 3μg or pcDNA-Empty 3μg). After 16 hrs cells were split into a 15cm dish and incubated overnight. Cells were then lysed in 3 mL denaturing buffer (8M Urea, 100 mM Tris pH 8, 40 mM imidazole, 500 mM NaCl, 1mM DTT, 2% NP-40/IGEPAL, 10% (v/v) ethylene glycol), sonicated and centrifuged at maximum speed for 15 minutes. His-tagged ubiquitin conjugates were then isolated using nickel-sepharose 6 resin (100 μL slurry). Lysates were incubated with the nickel-sepharose for 3 hrs by end over end rotation at 4^o^C, copiously washed (3 × 1mL lysis buffer as above and then 3 × 1mL of a solution containing 50mM Tris, 500mM NaCl, 40 mM imidazole, 1% (v/v) ethylene glycol). Samples were then eluted with 200 μL of buffer containing 300 mM imidazole, 50 mM Tris pH 8, 500 mM NaCl, 10% (v/v) ethylene glycol. 1 mM EDTA and 1mM DTT was added to the eluates which were then quantified by Bradford assay. Samples were loaded (100 μg each) onto a 7% SDS-PAGE gel. After electro-blotting onto nitrocellulose, the membrane was probed with the indicated antibodies.

### Notch assays

HEK-293T cells were transduced with a Cignal RBP-Jk Reporter (luc) Kit: CCS-014L (SABiosciences, UK.). After puromycin selection, pooled stable reporter cells were transfected with siRNA pools targeting CYLD (OpenBiosystems). Negative controls (non-targeting siRNA, siCON) were included. Transfected cells were then split after 12 hrs and re-seeded at sub-confluent levels. After 48 hrs the Notch (NICD) induced luciferase signal was quantified (One-Glo, Promega) and the results were normalized to cell viability (Cell-Titer Glo, Promega) in replicate wells and then to the siCON signal.

### Immunohistochemistry

Tissue sections from the TMA were dewaxed with xylene and rehydrated in graded ethanols, before undergoing microwave antigen retrieval in sodium citrate buffer (pH 6.0). Tissue sections were then blocked with peroxidase blocking agent (DAKO) and then incubated with the HES1, RUNX1 and LEF1 primary antibodies (Cell Signalling). Tissue sections were then washed in phosphate buffered saline and probed with secondary HRP-conjugated antibodies, and staining was visualized with 3, 3′-Diaminobenzidine (DAB). Haematoxylin was used as a nuclear counterstain.

### Microarray profiling

Genome-wide transcriptomic profiles of 32 CYLD defective tumours were generated, using RNA extracted from fresh frozen microdissected tissue as previously described [41]. In brief, a bead microarray platform was used to obtain the gene expression values from 32 tumour samples and 10 perilesional controls. Control tissue consisted of epidermis, hair follicles and associated appendageal structures. This platform, Illumina WG-DASL (San Diego, USA; http://www.illumina.com), allowed for monitoring of gene expression of 24, 526 transcripts. 50ng of total RNA from each sample was converted to cDNA as per the supplied protocol and then hybridised to Illumina Human 8v3 chips. A confocal Illumina Infinium bead reader then scanned the chips and the signal values were extracted using Illumina Beadstudio software 3.0. The transcriptomes are accessible at www.ebi.ac.uk at accession number E-MTAB-352.

### Quantitative PCR

Total RNA was extracted from cylindroma tumours as described previously [41], subject to DNAse digestion and then reverse transcribed using a Superscript III kit (Invitrogen, UK) according to manufacturers instructions. cDNA was then used for quantitative PCR with the following Taqman real time PCR primers HEY 1 (Hs01114113_m1), Beta –Actin - ACTB (Hs99999903_m1) with a Taq polymerase master mix (ABI, UK), in an ABI 7900HT thermal cycler. Gene expression was normalised to ACTB and comparisons in expression were made using the 2^−**ΔΔ**^Ct^^ formula as the PCR reactions had similar efficiencies of amplification.

### Protein expression quantification

5 representative immunohistochemistry images were taken from each tumour sample and control areas using a Zeiss Axioimager (Zeiss, UK). Image J software (v 1.48g – http://imagej.nih.gov/ij)) was used to quantify DAB staining. RGB values that were restricted to the colour of DAB in our staining protocol were used to select for areas in images that were DAB positive. The area of the image positive was then quantified and compared to the total area assessed to generate a ratio of positive staining. The mean of the ratios was used to generate a ratio per sample. The values in the tumours were then compared with the values in the controls using a t-test.

### Primary culture of CYLD defective tumour explants, HaCAT cells and drug experiments

Primary culture of cells derived from fresh tumour tissue was carried out as previously described on 3D tissue culture scaffolds [30]. After 28 days in culture, these scaffolds were grown for a further 12 days in the following γ-secretase inhibitors (Tocris Bioscience, UK) or control. HaCats were grown on tissue culture scaffolds and then cultured in identical conditions for 12 days. YO-0107 and Ly 450139 (Semagacestat) were both diluted in DMSO and the final concentration of DMSO in these and in the drug free controls was 1%.

## SUPPLEMENTARY INFORMATION, TABLE AND FIGURES


