# Supplementary material for: The cylindromatosis gene product, CYLD, interacts with MIB2 to regulate Notch signalling
Source: Oncotarget. 2014 Nov 3;5(23):12126–40. doi: 10.18632/oncotarget.2573 (PMC4322962; doi:10.18632/oncotarget.2573)
Supplement: Supplementary file 1 [file oncotarget-05-12126-s001.pdf]

SUPPLEMENTARY INFORMATION, TABLE AND FIGURES

Supplementary Table 1 Identification of MIB2 - CYLD co-precipitation by in-gel tryptic digest and tandem MS analysis:, ProFound Search Engine, Rank 1 Candidate, Accession AAH16490.1 (Uniprot Q96AX9-11;NP_543151.2), 'MIB2 Protein', Measured Peptides: 75, Matched peptides: 12, Minimum sequence coverage: 13%, Probability: 0.81. Peptides identified in the table are shown highlighted in gray within the MIB2 FASTA sequence below the table.

Supplementary Table S1. List of proteomic hits identified from mass spectrometric data. Identification of MIB2 - CYLD co-precipitation by in-gel tryptic digest and tandem MS analysis:, ProFound Search Engine, Rank 1 Candidate, Accession AAH16490.1 (NP_543151.2), 'MIB2 Protein', Measured Peptides: 75, Matched peptides: 12, Minimum sequence coverage: 13%, Probability: 0.81.

Measured Mass$_{AV}$	Calculated Mass	Residues	Missed Cuts	Peptide Sequence
868.604	868.524	17–24	1	VAPRSLAR
899.558	899.518	698–705	1	ARQLVDAK
927.556	927.499	61–68	1	RATGRPDR
1010.602	1010.587	315–323	0	LGKPAELQR
1027.595	1027.529	847–856	0	SPLDLAAEGR
1089.584	1089.534	21–30	1	SLARCGPSSR
1114.631	1114.634	411–420	1	VIGDLDTVKR
1210.788	1210.703	511–522	0	LVVEVALGNAAR
1282.714	1282.735	723–733	1	EVAQILIREGR
1461.768	1461.703	361–373	0	MAEFIGQTGTVHR *
1844.129	1843.953	949–967	0	LRPDGSEVASAAPAPGPPR
2224.963	2225.031	69–89	1	SRAAPPNMDPDPQAGVQVGMR**
				*(1)+ oxidised M, ** (2)+ oxidised M

MIB2 (Uniprot Q96AX9–11)

MAGALRRGRALGSRPSGPTVSSRRSPQCPVAQEGLGARSR PRVAPRSLAR CGPSSRLMGWKPSEARGQSQSFQ
ASGLQPRSLKAARRATGRPDR|SRAAPPNMDPDPQAGVQVGMRVVRGVDWNWGQQDGGEGGVGTVVELGRHGS
PSTPDRTVVVQWDQGTRTNYRAGYQGAHDLLLYDNAQIGVRHPNIICDCCKKHGLRGMRWKCRVCLDYDLCTQC
YMHNKHELAHAFDRYETAHSRPVTLSPRQGLPRIPLRGIFQGAKVVRGPDWEWGSQDGGEGKPGRVVDIRGWDVE
TGRSVASVTWADGTTNVYRVGHKGKVDLKCVGEAAGGFYYKDHLPRLGKPAELQRRVSADSQPFQHGDKVKCLLD
TDVLREMQEGHGGWNPRMAEFIGQTGTVHRITDRGDVRVQFNHETRWTFHPGALTKHHSFWVGDVVRVIGDLDTV
KRLQAGHGEWTDDMAPALGRVGKVVKVFGDGNLRVAVAGQRWTFSPSCLVAYRPEEDANLDVAERARENKSSLSV
ALDKLRAQKSDPEHPGRLVVEVALGNAARALDLLRRRPEQVDTKNQGRTALQVAAYLGQVELIRLLLQARAGVDL
PDDEGNTALHYAALGNQPEATRVLLSAGCRADAINSTQSTALHVAVQRGFLEVVRALCERGCDVNLPDAHSDTPLHS
AISAGTGASGIVEVLTEVPNIDVTATNSQGFTLLHHASLKGHALAVRKILARARQLVDAKKEDGFTALHLAALNNHRE
VAQILIREGRCDVNVRNRKLQSPLHLAVQQAHVGLVPLLVDAGCSVNAEDEEGDTALHVALQRHQLLPLVADGAGG
DPGPLQLLSRLQASGLPGSAELTVGAAVACFLALEGADVSYTNHRGRSPLDLAAEGRVLKALQGCAQRFRERQAGG
GAAPGPRQTLGTPNTVTNLHVGAAPGPEAAECLVCSELALLVLFSPCQHRTVCEECARRMKKCIRCQVVVSKKLRP
DGSEVASAAPAPGPPRQLVEELQSRYRQMEERITCPICIDSHIRLVFQCGHGACAPCGSALSACPICRQPIRDRIQIFV

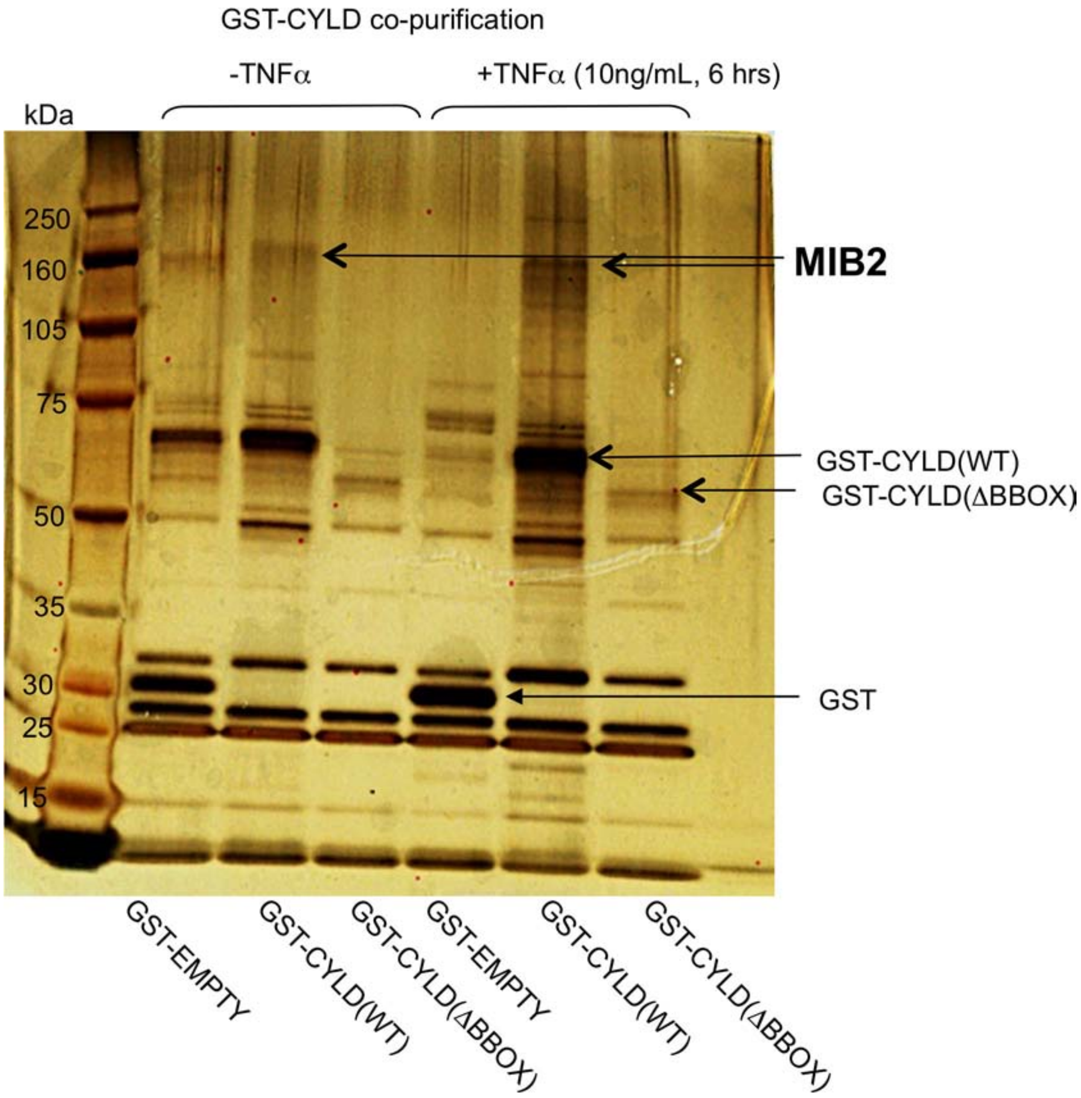

Supplementary Figure S1: GST pulldown assays performed in the presence and absence of TNF-alpha. MIB2 (arrowed) interacts with full length CYLD (GST-CYLD(WT)) in GST pull down assays, carried out both in the absence and presence of TNF-alpha. A CYLD construct mutated such that it did not contain a B Box domain (GST-CYLD(DBBOX)) [38], was also expressed in cells both in the presence and absence of TNF-alpha.

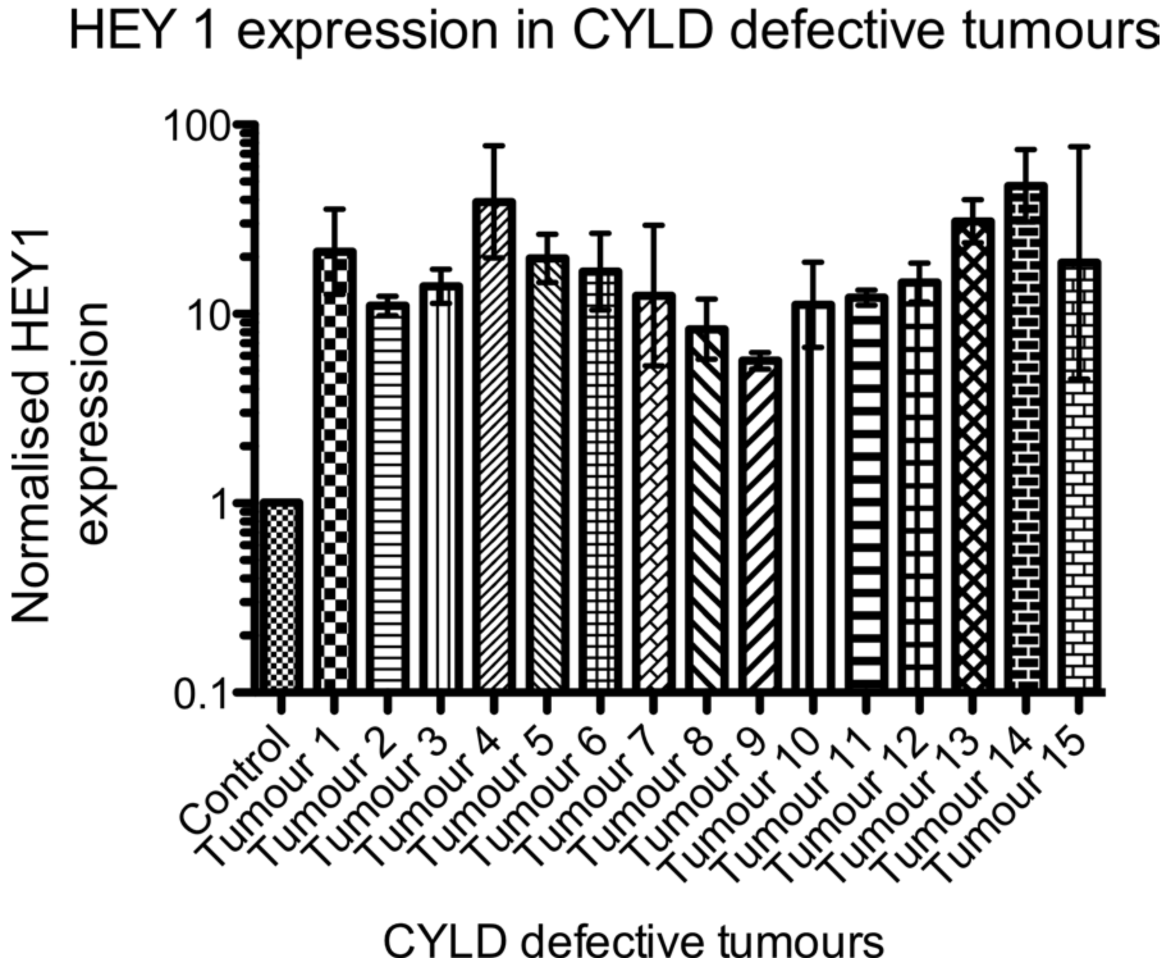

Supplementary Figure S2: HEY1 expression in CYLD defective tumours. Increased expression of HEY1 transcript was demonstrated in 15 CYLD defective tumours. RNA was extracted from microdissected, snap-frozen tumour tissue and subject to quantitative PCR using probes for HEY1. Beta-actin expression was also assessed and used to normalise expression levels. Differential expression was calculated using the 2 $^{(-\Delta\Delta Ct)}$ formula.
